# Supplementary material for: Does CytoSorb Interfere with Immunosuppression? A Pharmacokinetic and Functional Evaluation
Source: Pharmaceutics. 2025 Nov 13;17(11):1468. doi: 10.3390/pharmaceutics17111468 (PMC12655457; doi:10.3390/pharmaceutics17111468)
Supplement: Supplementary file 1 [file pharmaceutics-17-01468-s001.zip › Figure S1.pdf]

Supplementary Material

Does CytoSorb Interfere with Immunosuppression? A Pharmacokinetic and Functional Evaluation

Stephan Harm [1,*], Claudia Schildböck [1], Denisa Cont [1], Viktoria Weber [1], Jens Hartmann [1]

[1]*Department for Biomedical Research, University for Continuing Education Krems, Austria*

[*] **Correspondence:**

Stephan Harm

stephan.harm@donau-uni.ac.at

Keywords: **cytokines, CytoSorb, transplantation, immunosuppressive, hemoadsorption, hemodialysis, protein binding**

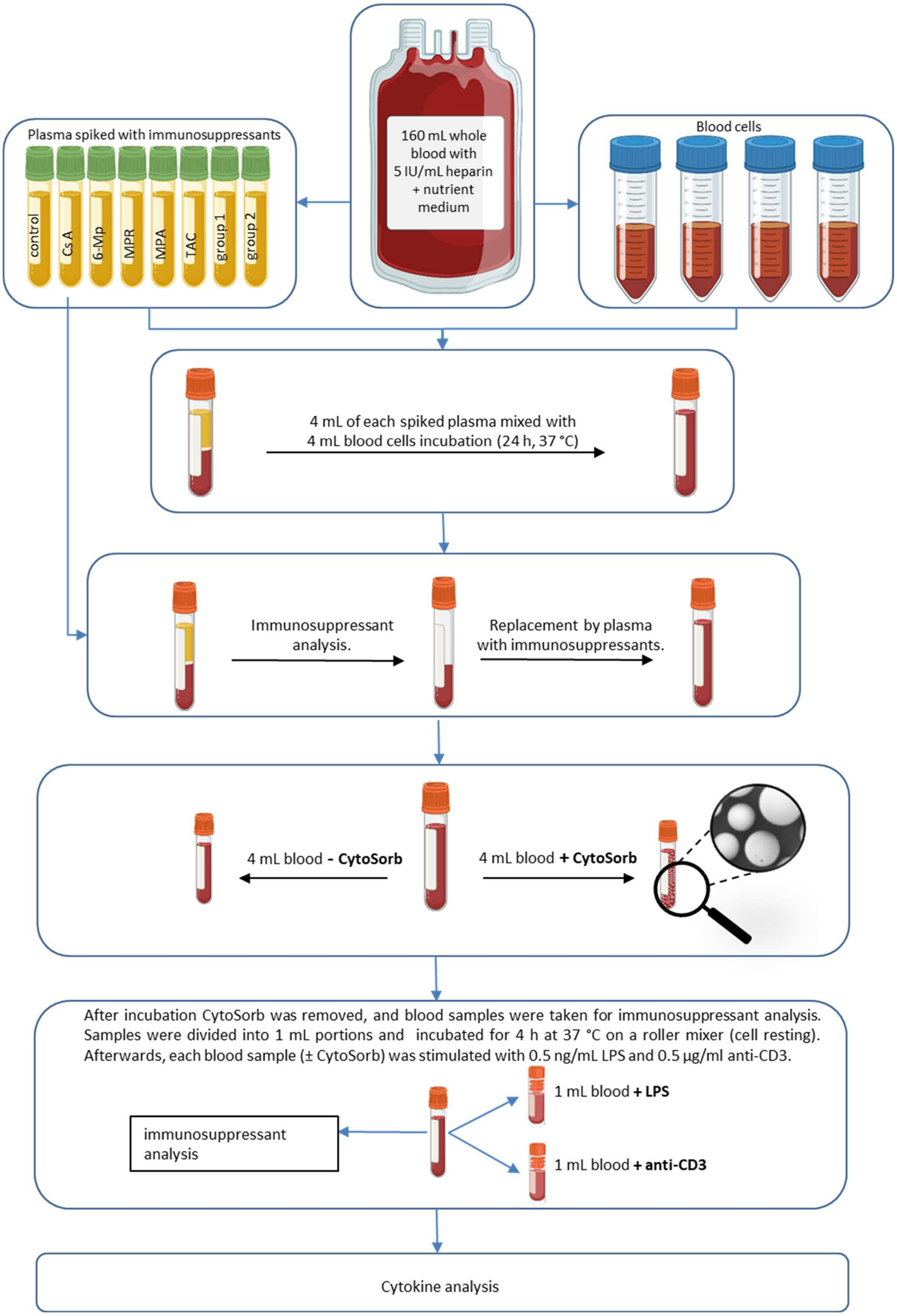
Plasma spiked with immunosuppressants
control
Cs A
6-Mp
MPR
MPA
TAC
group 1
group 2
160 mL whole blood with 5 IU/mL heparin + nutrient medium
Blood cells
4 mL of each spiked plasma mixed with 4 mL blood cells incubation (24 h, 37 °C)
Immunosuppressant analysis.
Replacement by plasma with immunosuppressants.
4 mL blood - CytoSorb
4 mL blood + CytoSorb
After incubation CytoSorb was removed, and blood samples were taken for immunosuppressant analysis. Samples were divided into 1 mL portions and incubated for 4 h at 37 °C on a roller mixer (cell resting). Afterwards, each blood sample (± CytoSorb) was stimulated with 0.5 ng/mL LPS and 0.5 µg/ml anti-CD3.
1 mL blood + LPS
immunosuppressant analysis
1 mL blood + anti-CD3
Cytokine analysis

Supplementary Figure 1: A blood cell model was established to determine the effect of a treatment with CytoSorb on immunosuppression. The immunosuppressants were tested individually and in groups. Treatment was performed after overnight incubation. The leukocytes were separately stimulated with LPS and anti-CD3. After 4 h of incubation, the cytokine secretion was measured and compared with stimulated blood without immunosuppressants.
