# Supplementary material for: Does CytoSorb Interfere with Immunosuppression? A Pharmacokinetic and Functional Evaluation
Source: Pharmaceutics. 2025 Nov 13;17(11):1468. doi: 10.3390/pharmaceutics17111468 (PMC12655457; doi:10.3390/pharmaceutics17111468)
Supplement: Supplementary file 1 [file pharmaceutics-17-01468-s001.zip › Table S1.pdf]

Supplementary Material

Does CytoSorb Interfere with Immunosuppression? A Pharmacokinetic and Functional Evaluation

Stephan Harm [1,*], Claudia Schildböck [1], Denisa Cont [1], Viktoria Weber [1], Jens Hartmann [1]

[1]*Department for Biomedical Research, University for Continuing Education Krems, Austria*

[*] Correspondence:

Stephan Harm

stephan.harm@donau-uni.ac.at

Keywords: **cytokines, CytoSorb, transplantation, immunosuppressive, hemoadsorption, hemodialysis, protein binding**

Supplementary Table 1

Cytokine serum levels of the individual whole blood stimulation experiments. The positive control was LPS and anti-CD3 activated blood from the same donor without immunosuppressants. Unstimulated blood without immunosuppressants served as negative control.

Cyclosporin A

	test	***IL-1β* [pg/mL]**	***IL-6* [pg/mL]**	***IL-8* [pg/mL]**	***TNF-α* [pg/mL]**
+ LPS - CytoSorb	1	208	2801	5649	2978
	2	75	546	2382	1273
	3	712	3075	1762	1189
+ LPS + CytoSorb	1	167	2440	4280	2442
	2	72	632	1762	1189

	3	358	18993	19679	1345
+ Anti-CD3 - CytoSorb	1	0	5	424	8
	2	178	2079	13867	66
	3	263	3075	20120	90
+ Anti-CD3 + CytoSorb	1	0	3	205	12
	2	72	632	1762	46
	3	140	857	5980	57

6-Mercaptopurine

	test	*IL-1β* [pg/mL]	*IL-6* [pg/mL]	*IL-8* [pg/mL]	*TNF-α* [pg/mL]
+ LPS - CytoSorb	1	302	4450	8490	3711
	2	58	543	3719	1902
	3	326	5714	12110	1961
+ LPS + CytoSorb	1	354	3786	6337	2920
	2	44	730	2253	1762
	3	190	3799	7104	1250
+ Anti-CD3 - CytoSorb	1	0	7	1526	53
	2	178	2079	13867	66
	3	263	3075	20120	90
+ Anti-CD3 + CytoSorb	1	0	3	205	12
	2	75	381	4175	46
	3	70	30	686	41

Methylprednisolone

	test	*IL-1β* [pg/mL]	*IL-6* [pg/mL]	*IL-8* [pg/mL]	*TNF-α* [pg/mL]
+ LPS - CytoSorb	1	144	1341	4361	2292
	2	29	37	1522	435
	3	207	941	5713	1127
+ LPS + CytoSorb	1	354	3786	6337	2920
	2	26	73	1630	416
	3	190	3799	7104	1250
+ Anti-CD3 - CytoSorb	1	0	7	1526	53
	2	42	18	322	44
	3	62	23	322	41

+ Anti-CD3 + CytoSorb	1	0	1	171	43
	2	44	16	242	57
	3	70	30	686	41

Mycophenolate

	test	***IL-1β*** **[pg/mL]**	***IL-6*** **[pg/mL]**	***IL-8*** **[pg/mL]**	***TNF-α*** **[pg/mL]**
+ LPS - CytoSorb	1	482	1696	4151	3145
	2	138	603	3212	1491
	3	2615	19976	23825	1182
+ LPS + CytoSorb	1	992	3303	4068	3390
	2	112	627	2855	1274
	3	1465	16855	15529	720
+ Anti-CD3 - CytoSorb	1	18	4	1606	145
	2	191	1134	7046	144
	3	265	1676	8832	68
+ Anti-CD3 + CytoSorb	1	0	4	564	107
	2	115	645	3704	109
	3	170	952	4924	55

Tacrolimus

	test	***IL-1β*** **[pg/mL]**	***IL-6*** **[pg/mL]**	***IL-8*** **[pg/mL]**	***TNF-α*** **[pg/mL]**
+ LPS - CytoSorb	1	473	8969	10664	2471
	2	96	562	3353	1767
	3	933	39226	75450	1785
+ LPS + CytoSorb	1	412	7095	7756	1967
	2	62	584	2388	1337
	3	646	23302	50044	690
+ Anti-CD3 - CytoSorb	1	21	113	2572	23
	2	225	1749	20222	82
	3	313	2478	27385	99
+ Anti-CD3 + CytoSorb	1	18	28	1654	38
	2	93	773	8613	53
	3	268	2598	25920	85

Group 1

	test	*IL-1β* [pg/mL]	*IL-6* [pg/mL]	*IL-8* [pg/mL]	*TNF-α* [pg/mL]
+ LPS - CytoSorb	1	392	12062	15405	3274
	2	52	578	2454	1132
	3	343	18712	27279	1405
+ LPS + CytoSorb	1	270	7114	7298	2192
	2	37	367	1689	733
	3	301	12960	15732	1212
+ Anti-CD3 - CytoSorb	1	52	121	3339	30
	2	160	761	11581	67
	3	185	1007	13819	69
+ Anti-CD3 + CytoSorb	1	0	14	582	7
	2	58	140	1875	35
	3	86	193	2195	45

Group 2

	test	*IL-1β* [pg/mL]	*IL-6* [pg/mL]	*IL-8* [pg/mL]	*TNF-α* [pg/mL]
+ LPS - CytoSorb	1	988	13184	19861	2283
	2	120	174	1580	391
	3	331	1261	5542	997
+ LPS + CytoSorb	1	189	2024	4379	777
	2	55	141	1068	292
	3	157	199	2039	336
+ Anti-CD3 - CytoSorb	1	11	87	2207	18
	2	51	75	1570	39
	3	65	23	118	40
+ Anti-CD3 + CytoSorb	1	42	29	648	57
	2	76	35	484	65
	3	70	23	69	39

negative controls (without immunosuppressants)

	test	*IL-1β* [pg/mL]	*IL-6* [pg/mL]	*IL-8* [pg/mL]	*TNF-α* [pg/mL]
no stimulation	1	60	995	4823	28
	2	0	0	0	0

	3	255	7346	6281	79

positive controls (without immunosuppressants)

	test	***IL-1β* [pg/mL]**	***IL-6* [pg/mL]**	***IL-8* [pg/mL]**	***TNF-α* [pg/mL]**
+ LPS - CytoSorb	1	2856	30759	32188	4097
	2	135	622	3144	1280
	3	786	26043	36096	1052
+ LPS + CytoSorb	1	445	9237	10747	2531
	2	63	449	1792	1071
	3	387	18195	19053	829
+ Anti-CD3 - CytoSorb	1	606	3912	20781	118
	2	664	4334	32858	140
	3	378	2509	27899	90
+ Anti-CD3 + CytoSorb	1	62	48	2683	60
	2	132	524	6711	81
	3	133	729	7260	60
